# Evaluation of BLAST-based edge-weighting metrics used for homology inference with the Markov Clustering algorithm

**DOI:** 10.1186/s12859-015-0625-x

**Published:** 2015-07-10

**Authors:** Theodore R. Gibbons, Stephen M. Mount, Endymion D. Cooper, Charles F. Delwiche

**Affiliations:** 1 Department of Cell Biology and Molecular Genetics, University of Maryland, College Park, Baltimore, Maryland 20742 USA; 2 Center for Bioinformatics and Computational Biology, University of Maryland, College Park, Baltimore, Maryland 20742 USA; 3 Maryland Agricultural Experiment Station, University of Maryland, College Park, Baltimore, Maryland 20742 USA

**Keywords:** MCL, Protein clustering, Sequence clustering, Homology prediction, Graph, Genomics, Bioinformatics, Transcriptomics, Short-read sequencing, High-throughput sequencing

## Abstract

**Background:**

Clustering protein sequences according to inferred homology is a fundamental step in the analysis of many large data sets. Since the publication of the Markov Clustering (MCL) algorithm in 2002, it has been the centerpiece of several popular applications. Each of these approaches generates an undirected graph that represents sequences as nodes connected to each other by edges weighted with a BLAST-based metric. MCL is then used to infer clusters of homologous proteins by analyzing these graphs. The various approaches differ only by how they weight the edges, yet there has been very little direct examination of the relative performance of alternative edge-weighting metrics. This study compares the performance of four BLAST-based edge-weighting metrics: the bit score, bit score ratio (BSR), bit score over anchored length (BAL), and negative common log of the expectation value (NLE). Performance is tested using the Extended CEGMA KOGs (ECK) database, which we introduce here.

**Results:**

All metrics performed similarly when analyzing full-length sequences, but dramatic differences emerged as progressively larger fractions of the test sequences were split into fragments. The BSR and BAL successfully rescued subsets of clusters by strengthening certain types of alignments between fragmented sequences, but also shifted the largest correct scores down near the range of scores generated from spurious alignments. This penalty outweighed the benefits in most test cases, and was greatly exacerbated by increasing the MCL inflation parameter, making these metrics less robust than the bit score or the more popular NLE. Notably, the bit score performed as well or better than the other three metrics in all scenarios.

**Conclusions:**

The results provide a strong case for use of the bit score, which appears to offer equivalent or superior performance to the more popular NLE. The insight that MCL-based clustering methods can be improved using a more tractable edge-weighting metric will greatly simplify future implementations. We demonstrate this with our own minimalist Python implementation: Porthos, which uses only standard libraries and can process a graph with 25 m + edges connecting the 60 k + KOG sequences in half a minute using less than half a gigabyte of memory.

**Electronic supplementary material:**

The online version of this article (doi:10.1186/s12859-015-0625-x) contains supplementary material, which is available to authorized users.

## Background

Clustering protein sequences by inferred homology (descent from a common ancestral sequence) is a fundamental step for many analyses involving the growing number of large sequence data sets. Functional and structural predictions may be greatly accelerated by analyzing only a single representative sequence from each cluster and then transferring these annotations to the other cluster members. Other methods, such as phylogenetic inference, can only be applied to individual homologous groups. In either case, errors introduced in this critical early step can propagate throughout downstream analyses. It is therefore imperative that the sources and causes of these errors are understood so that they can be avoided, or at least mitigated.

Perhaps the most popular approach for identifying homologous sequences shared between multiple genomes is to generate all against all pairwise alignments using a program such as BLAST [1, 2], followed by the application of fixed filtering thresholds or the Reciprocal Best Hits (RBH) algorithm [3]. Fixed filtering thresholds can be an efficient way to remove large numbers of very poor hits, but are ineffective for most other uses [4]. RBH works well for analyzing pairs of proteomes containing few inparalogs or other recently duplicated protein-coding genes, but is not easily extended to more complex cases that violate these conditions (we follow here the molecular homology terminology of Sonnhammer and Koonin [5-7]). Some attempts have been made to extend RBH for specific situations [5, 8], but the algorithm is not easily generalizable.

The Markov Cluster (MCL) algorithm (and corresponding program of the same name) is a robust and widely used alternative to RBH [9-11]. It was designed to handle data from an arbitrary number of organisms whose proteins share arbitrarily complex evolutionary histories. MCL operates on an abstracted graph-representation of a set of BLAST hits in which each sequence is stored as a node, and each BLAST hit is stored as a weighted edge connecting a pair of sequences. At the time of its release, the MCL program was unable to directly read a table of BLAST hits, and so the TribeMCL Perl module was published alongside the original MCL program to handle this task [9]. The following year, the OrthoMCL suite of Perl scripts extended the TribeMCL method by normalizing the edge weights using inter-organism averages, while simultaneously circumventing memory limitations by interfacing with a MySQL relational database [10].

It has now been over a decade since the original publication of these programs, and the typical computer workstation contains multiple processing cores and has significantly more memory. These improvements in hardware recently motivated another group to reimplement OrthoMCL as a standalone, multithreaded C++ program orthAgogue [11]. With it, Ekseth et al. also introduced an option to use the bit score as an alternative to the negative common log (−log_10_) of the expectation value (NLE) edge-weighting metric used by both TribeMCL and OrthoMCL. While the authors described several practical benefits of switching to the bit score, they offered no demonstration of its performance compared to the traditional NLE.

This study provides the first direct performance comparison between the NLE and BS. To avoid potential confounding effects introduced by heuristic discrepancies between different implementations, we wrote a custom version of OrthoMCL in Python. The program stores and processes all metrics identically, and outputs a set of graphs that differ only by their edge weights. Two additional metrics, not previously used for MCL-base clustering, were included: the bit score divided by either the self bit score (termed BLAST score ratio [12, 13] or bit score ratio; BSR) or the anchored alignment length (bit score over anchored alignment length; BAL). These latter two metrics were included to address fragmented and partial sequences that often result from short-read *de novo* DNA and RNA sequencing projects. Bit scores and E-values from alignments between these fragmented sequences can easily fall into the range of spurious hits between very distantly or unrelated sequences, causing the corresponding edges to be removed by MCL and thus leading to unwanted cluster fragmentation.

We compare here the performance of these four edge-weighting metrics over a range of MCL inflation parameter values, and consider different sequence fragmentation scenarios varying from all sequences being intact, to some or all being split into two or three subsequences. Contrary to our expectations, we observed that the bit score matched or exceeded the performance of all other edge-weighting metrics in each scenario. This suggests that the performance of the popular pipeline is actually improved by switching to the relatively simple bit score as an edge-weighting metric.

## Results and discussion

### Test database creation

Evaluation of inference methods requires a reference data set for which the correct solutions are known. It is not possible to go back and directly observe the evolution of extant species, and there is no single, universally recognized reference dataset for the problem of clustering protein sequences based on inferred homology, although the manually curated Eukaryotic Orthologous Groups (KOG) database is a popular choice [14]. For this study, we used an extension of the Conserved Eukaryotic Genes Mapping Approach (CEGMA) database [15], which is a subset of the KOG database.

The KOG database was derived from the proteomes of seven eukaryotes whose genomes had been sequenced, annotated, and published by 2003 (*Saccharomyces cerevisiae* [16], *Caenorhabditis elegans* [17], *Arabidopsis thaliana* [18], *Drosophila melanogaster* [19], *Encephalitozoon cuniculi* [20], *Homo sapiens* [21], and *Schizosaccharomyces pombe* [22]). Each KOG (cluster) is an assertion that the sequences within it share a more recent common ancestor with each other than with sequences in any other KOG, guaranteeing neither that a KOG is free of outparalogs, nor that it contains all members of a particular group of (co)orthologs. This is partially due to the consideration of functional data by the human curators, and because only 50-75 % of the predicted proteins from each organism were included. Despite this reduction from its potential size, the 60,758 KOG-annotated protein sequences still proved to be inconveniently large for the dozens of all vs. all BLASTP jobs required for this study.

The CEGMA database is a more computationally tractable alternative, containing sequences from only 458 KOGs that span all six free-living eukaryotes [15]. From these KOGs, however, the CEGMA developers removed all sequences from the parasite *E. cuniculi*, as well as inparalogous sequences, which we observed to be a major source of false negative errors when clustering sequences based on inferred homology. Restoring these sequences to the 458 CEGMA KOGs also restored the false negative errors observed when analyzing the complete KOG database (Additional File 1: Figure S1). The addition of sequences from four other eukaryotes (*Anopheles gambiae* [23], *Ciona intestinalis* [24], *Chlamydomonas reinhardtii* [25], and *Toxoplasma gondii* [26]), for which the CEGMA developers have provided annotations, led to a test data set we refer to as the Expanded CEGMA KOGs (ECK) database. ECK has been used for all tests in this study, except where otherwise noted. Statistics for all three databases are shown in Table 1.

**Table 1:** Statistics for the KOG, CEGMA, and ECK databases. The Eukaryotic Orthologous Groups (KOG) database contains sequences from seven eukaryotic genomes that were available at the time of its creation in 2003. The Conserved Eukaryotic Genes Mapping Approach (CEGMA) database is a subset of 458 KOGs that contain at least one sequence from each of the six free-living KOG organisms, from which inparalogs were then removed. These inparalogs were restored in the Expanded CEGMA KOGs (ECK) clusters, and sequences from four additional taxa annotated by the CEGMA developers were added

	KOG		CEGMA		ECK	
	Seqs	KOGs	Seqs	CEGs	Seqs	ECKs
*Homo sapiens*	19,039	4,597	458	458	1,350	458
*Arabidopsis thaliana*	13,744	3,285	458	458	1,175	458
*Caenorhabditis elegans*	10,581	4,235	458	458	635	458
*Drosophila melanogaster*	8,445	4,351	458	458	611	458
*Saccharomyces cerevisiae*	4,003	2,668	458	458	606	458
*Schizosaccharomyces pombe*	3,728	2,762	458	458	557	458
*Encephalitozoon cuniculi*	1,218	1,073	-	-	311	291
*Anopheles gambiae*	-	-	-	-	453	453
*Ciona intestinalis*	-	-	-	-	432	432
*Chlamydomonas reinhardtii*	-	-	-	-	407	407
*Toxoplasma gondii*	-	-	-	-	303	303
Database Totals	60,758	4,852	2,748	458	6,840	458

### Software implementation

Each implementation of the TribeMCL BLAST-graph clustering algorithm uses custom software to convert a table of BLASTP hits into a MCL-readable graph. Complex software inevitably contains unique elements that can lead to performance differences between implementations of the same algorithm. Most of these differences will be slight and arise from seemingly minor decisions, such as whether to use the larger score from a pair of reciprocal BLAST hits (which are typically very similar, but occasionally unequal), or to average the two. It is difficult to catalogue these differences across published implementations, and harder still to gauge their impact. To remove such confounding factors from our comparison of the performance of different edge-weighting metrics, we wrote our own implementation, which stores all competing metrics within a single data structure and acts on them identically. It then prints a set of topologically identical graphs, differing only by edge weights.

Recent publications have focused on improving the computational performance in the graph creation [11] and clustering steps [27]. However, in our tests, the runtime of every approach has been dominated by the shared BLASTP step. Even our unoptimized Python implementation required only a small fraction of the CPU cycles of BLASTP, and used only a few hundred megabytes of memory for the complete 60 k + sequence KOG database. Our implementation, along will all other scripts used in our analysis pipeline, are publicly available at https://github.com/trgibbons/BlastGraphMetrics. This software was not intended for use beyond this study, so we also developed a more user-friendly implementation of the blast2graphs.py program called Porthos, which includes a basic version relying only on standard Python libraries. Our hope is that Porthos will alleviate many challenges associated with software installation, and that the code may serve as a helpful guide for future reimplementations. Porthos can be found at https://github.com/trgibbons/porthos.

### BLAST graph topology and E-value cutoff

All edge-weighting metrics used in this study are derived from only the top scoring hit between each pair of sequences within a given test database. The theoretical limit for the number of such top hits is the square of the number of sequences in the database, although in practice the number of BLAST hits that pass any reasonably stringent E-value threshold will be much less than this theoretical limit. Consequently, each BLAST graph passed to MCL will be sparse, comprised of connected components that share no edges between them. Each of these connected components can be evaluated by the same criteria used to evaluate the clusters output by MCL, providing a baseline for the performance of MCL using any metric for weighting the edges of the adjacency graph.

**Fig. 1:**
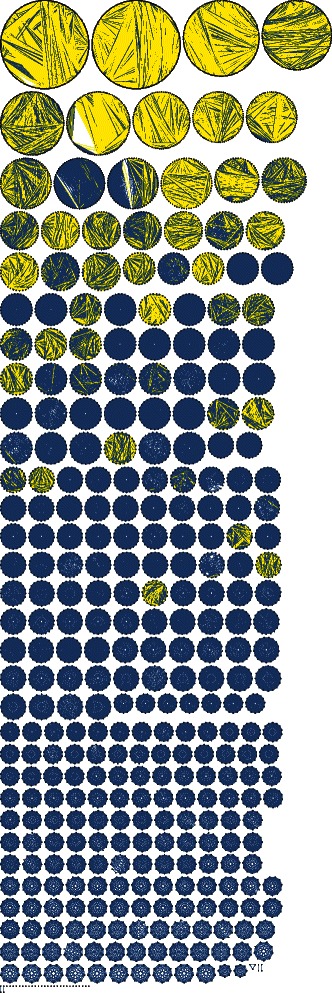
Pre-clustering BLAST graph for ECK database with E-value ≤ 1e-5. BLAST graph representing sequences as nodes that have been arranged into rings corresponding to the connected components. Blue edges connect sequences from the same KOG. Yellow edges connect sequences from different KOGs. The blue circles represent single-KOG clusters that have been successfully resolved using only BLAST’s E-value threshold option (1e-5 in this case)

Figure 1 shows the topology of the BLAST graph for the ECK database using an E-value cutoff of 1e-5. Each connected component can be considered a BLAST cluster. Before subsequent clustering with MCL, 237/458 ECKs have been perfectly reconstructed (contain all members of exactly one ECK) using BLASTP alone. Thirty one ECKs were split into multiple BLAST clusters and therefore cannot be rescued by MCL, which will divide connected components, but never combine them. In the end, only 54 BLAST clusters stand to be improved by MCL. The goal at this stage is to choose an edge-weighting metric that will give MCL the greatest chance to resolve or improve these multi-ECK BLAST clusters without (further) incorrectly splitting the 291 single-ECK BLAST clusters.

Predictably, a cutoff of 1e-3 decreased the number of fragmented ECKs and increased the average size of multi-ECK clusters, although it had little effect on the number of perfectly resolved ECKs (239 vs 237). The effects of the E-value threshold on sequence clustering have previously been addressed in some detail [28, 29], so we chose not to explore this further and instead settled on a fixed threshold of 1e-5.

### Simulated sequence fragmentation

**Fig. 2:**
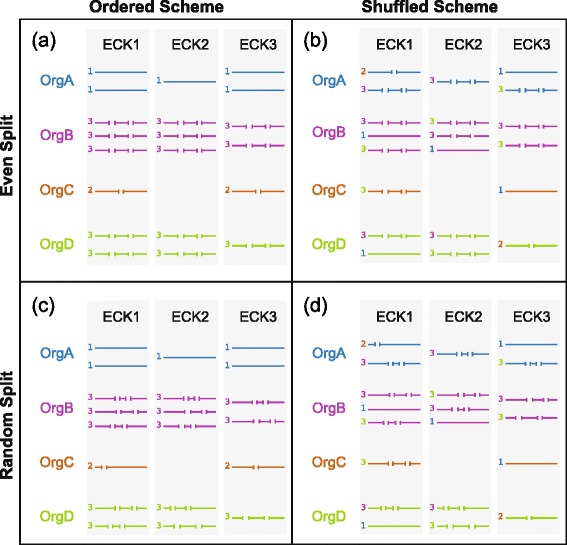
Illustration of simulated sequence fragmentation. This example illustrates the four different ways in which the fragmentation scheme 1323 would be applied to a toy input test database with only three clusters containing sequences from only four organisms. The four resulting test sets represent a cross of two variables, arranged here into rows and columns. The sequences in the top row (**a** & **b**) have been split into even subsequences. The sequences in the lower row (**c** & **d**) have been randomly fragmented into uneven subsequences. In the left column (**a** & **c**), the user-defined integer assigned to each organism directly determines the number of subsequences into which each sequence is split. In the right column (**b** & **d**), these integers are first mapped to all sequences within a cluster, but are then shuffled within that cluster before fragmentation

Part of the motivation for this study was the problem posed by fragmented sequences resulting from *de novo* transcript assembly. It is not possible to generate alignments that span the full-length of a protein sequence when part of the sequence is missing. Previous studies have not addressed the impact of this on either the weighted adjacency matrix or the resulting clusters. This study explored the effects of fragmentation on clustering by splitting portions of the sequences in the ECK database into two or three subsequences before performing the all-vs-all BLASTP alignments. To determine if sequences from different organisms played equivalent roles in cluster formation, each fragmentation scheme was first applied along organismal lines (Fig. 2, a & c), then randomly, such that a sequence’s organism of origin did not affect its likelihood of being fragmented into a particular number of subsequences (Fig. 2, b & d).

The number of sequences contributed from a particular organism varies within each ECK. To ensure that the relative proportions of sequence fragmentation remained constant within a given ECK across test scenarios, fragmentation labels were applied to each sequence within each ECK according to organismal origin, prior to fragmentation. One set of test sequences was generated by fragmenting them according to these labels (i.e., sequences labeled with a “2” were split once, those labelled with a “3” were split twice, and sequences labelled with a “1” were left intact), then the labels were shuffled and reapplied to obtain a second set of fragmented sequences.

Within these “ordered” and “shuffled” test sets, the effects of applying evenly spaced breakpoints (Fig. 2, a & b) were tested against the application of randomly spaced breakpoints (Fig. 2, c & d), resulting in a total of four test data sets for each fragmentation scheme. No differences were observed between the “ordered” and “shuffled” test sets for any fragmentation scenario (Additional file 1: Figures S2 & S3). In contrast, the distribution of break points within each sequence, and therefore often the alignment of break points across homologous sequences, had a dramatic effect in every test case (see following section).

### Edge-weighting metrics and sequence fragmentation

The transformation of a set of BLAST hits into an MCL-readable graph encompasses the majority of the steps that distinguish the popular software packages from one another and is at the heart of this study. TribeMCL, OrthoMCL, and later versions of MCL itself, all use the NLE from the top hit between each pair of sequences as the basis for the edge weights in the BLAST graph. In addition to the NLE, the recently published orthAgogue program also offers a sum of bit scores from all non-overlapping hits between two sequences as an alternative edge-weighting metric.

There are several practical benefits gained by using the bit score in place of the NLE [11], most notably the ability to combine scores from non-overlapping hits using simple addition. Another important benefit is that, while BLAST (v2.2.28+) rounds E-values below 1e-180 to zero, all bit scores are accurately reported. The log of zero is undefined and, so these “missing” values must be heuristically supplemented. Even alignments between protein sequences of modest length (ca. 200–300 amino acids) can generate E-values exceeding this rounding threshold, so this is not merely a theoretical concern. In practice, many of the strongest NLE edges in the graph end up being weighted by heuristics, rather than by the rigorous statistical metrics produced by BLAST. Using bitscores to weight the edges avoids this complication.

Use of the bit score introduces a different problem, however, which the BLAST E-value was meant to solve. The bit score is linearly correlated with alignment length. In fact, by definition the bit score increases, on average, by two *bits* of information for every pair of aligned amino acids. This means that long sequences have the ability to produce large bit scores from relatively poor alignments, while short sequences may not be able to generate large bit scores from even perfect, full-length alignments. The E-value attempts to account for these potential differences in sequence length (Equation 1), but (for the purposes of MCL-based clustering) does so at the cost of the other practical limitations mentioned above.

1 $$ \mathrm{E}-\mathrm{value}=\frac{mN}{2_{BS}}. $$

*m*= query sequence length

*N*= concatenated sequence database length

*BS*= bit score

In their original 2002 paper, Enright et al. mentioned that alternative metrics using length normalization might outperform the NLE. Until recently, this area has not received much attention in the literature. One reason may be that high quality alignments between pairs of full-length sequences are often (nearly) end-to-end. As long as detectable homologs are of similar lengths and are not fragmented, competing alignments will have both similar scores and lengths, negating any effects from length-based normalization. These assumptions are violated by partial and fragmented sequences, which are increasingly common as the products of high-throughput short-read *de novo* sequencing projects. It therefore seemed worth considering such metrics alongside the NLE and the BS.

**Fig. 3:**
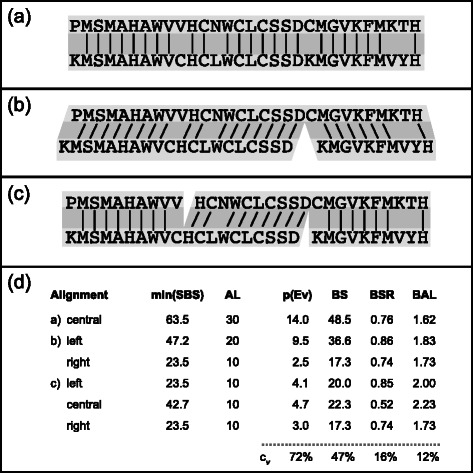
Illustration of edge-weighting metrics. Toy example demonstrating performance similarities and differences between the graph-weighting metrics in three different simulated fragmentation scenarios: (a) alignment between two full-length sequences, (b) alignment between one full-length sequence and one unevenly fragmented sequence, and (c) alignment between two unevenly fragmented sequences. Section (d) lists information about each alignment, including the minimum self bit score (SBS), the anchored alignment length (AL), and each of the four edge-weighting metrics. The coefficients of variation (c_v_ = σ/μ) summarize the variation relative to the respective means for each metric

Of the novel metrics considered for this study, the simplest is the bit score divided by the length of the shorter of the two sequences. This metric penalizes partial alignments between full-length sequences that share only a small conserved domain, while aiming to give equivalent weight to alignments between homologous sequences, whether they are both full-length (Fig. 3a), similarly fragmented, or one has been fragmented into a subsequence of the other (Fig. 3b). To work as intended, this metric assumes a direct linear relationship between alignment length and bit score. As stated above, the bit score between two perfectly aligned sequences will be twice the alignment length, on average, but the underlying distribution could be broad and/or skewed.

**Fig. 4:**
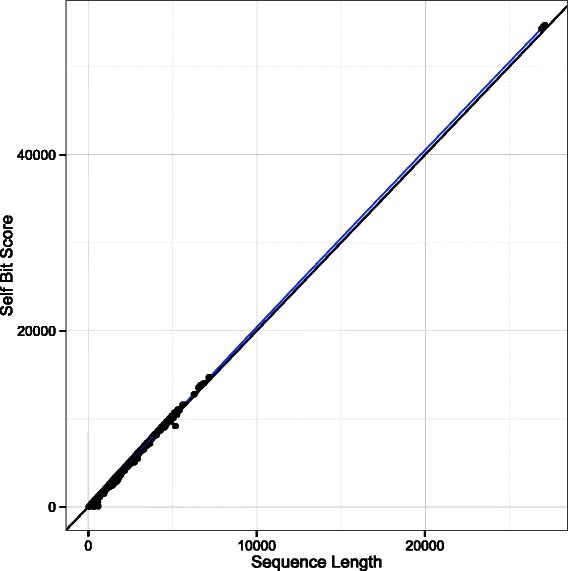
Self bit scores vs sequence lengths. Scatterplot of self bit score vs sequence length for all 60 k + protein sequences in the KOG database, plotted against the theoretical bit score = 2x sequence length (black line). The blue line is a smoothed line of best fit. 95 % confidence intervals were plotted, but are not visible due to the tightness of the distribution

To observe a real distribution, we plotted bit scores from full-length self-alignments (self bit score; SBS) against sequence lengths for the complete KOG database (Fig. 4). The correlation turned out to be remarkably tight and close to the theoretical relationship (blue line). Furthermore, preliminary results showed no appreciable difference between this simple bit score/length metric and a modified version using a ratio of the alignment bit score to the smaller of the two SBSs (bit score ratio; BSR), so only the results from the BSR are included here. Despite the similar performance, the BSR is preferable because it does not require modification of the tab-delimited BLAST output, which does not contain the sequence lengths by default, and because it remains theoretically possible for a sequence to generate a SBS substantially smaller or larger than twice its length.

As with simple length normalization, the BSR is designed to rescue edges between homologous sequences when one has been fragmented into a subsequence of the other, and it likewise falters when both sequences are incomplete and overlap on opposing ends (Fig. 3c, middle alignment). In these cases, dividing by the full length of the shorter of the two sequences (or the smaller of the two SBSs) penalizes alignments for not extending beyond the homologous region shared between the two sequences, and in this way does not faithfully accomplish what is desired with such normalization. Unfortunately, while bit scores can be easily combined, they are not easily split. Computing a SBS for just the alignable region would require re-running BLASTP after identifying a set of alignable homologous regions. It is therefore convenient that sequence length turned out to be a reasonable proxy for the SBS in most cases, as it is much more easily manipulated. These observations inspired the final metric considered in this study.

For the BAL metric, the aligned sequences are first anchored relative to each other based on the coordinates of the top BLASTP hit. The bit score from each alignment is then divided by the sum of the alignment length and the lengths of the shorter overhanging sequences extending from either side of the aligned region (Fig. 3). In other words, the bit score from each top hit is normalized by the length of the maximum alignable region anchored by that hit. This inflates edge weights between fragmented sequences, whether or not one is a subsequence of (a homolog of) the other, while simultaneously deflating edge weights between full-length sequences that share only a small conserved domain.

In practice, all three scenarios shown in Fig. 3 are likely to be encountered when clustering data from short-read *de novo* sequencing projects, and edges with relatively small weights will be eliminated in favor of those with larger weights. The coefficients of variation (a ratio of the standard deviation to the mean) illustrate how dramatically the BSR and BAL metrics can reduce the range of scores between competing high-quality alignments, increasing the likelihood that alignments between overhanging ends will persist and connect subclusters within a group of fragmented homologous sequences.

**Fig. 5:**
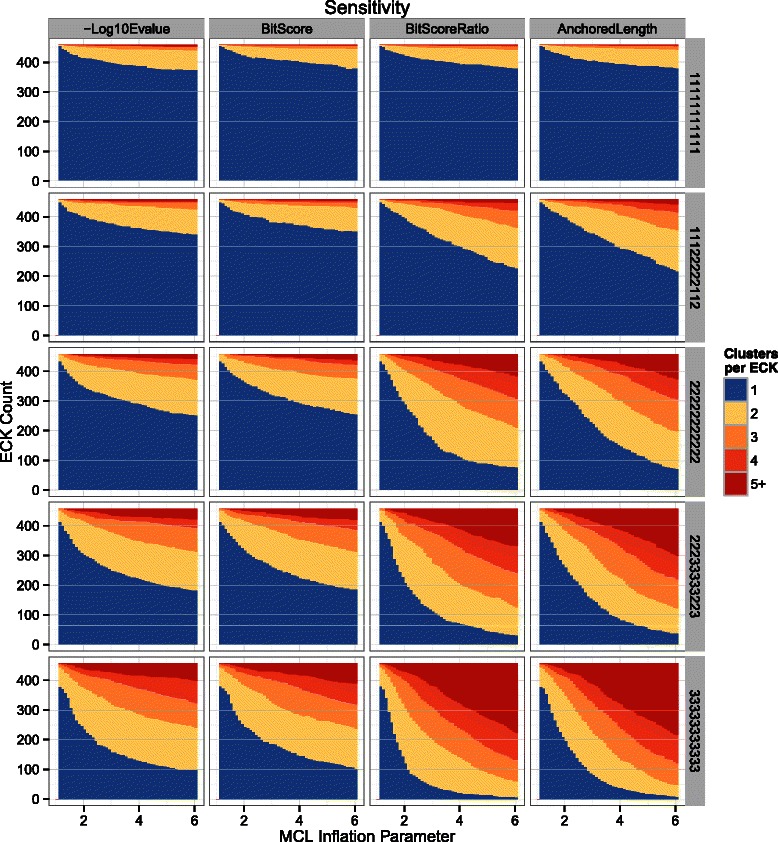
Sensitivity performance comparison for each edge-weighting metric and fragmentation scenario. Sensitivity performance of MCL on graphs weighted using each of the four metrics (columns) over a range of inflation parameter values (x-axes) in a variety of fragmentation scenarios (rows). Vertically stacked bars indicate the number of clusters into which the members of a particular ECK have been split. Blue segments represent ECKs that were completely contained within a single MCL cluster. Other segments represent ECKs that were split into two or more MCL clusters, with redder color indicating higher degrees of fragmentation. The number of ECKs is fixed, so each stack sums to exactly 458. Five different simulated fragmentation scenarios are displayed as faceted rows: 11111111111 – all sequences are intact; 11122222112 – approximately half of the sequences have been split into two pieces; 22222222222 – all sequences have all been split into two pieces; 33311222111 – approximately one third of the sequences have been split into two pieces, one third have been split into three pieces, and the remaining third were left intact; 33333333333 – all sequences were split into three pieces

**Fig. 6:**
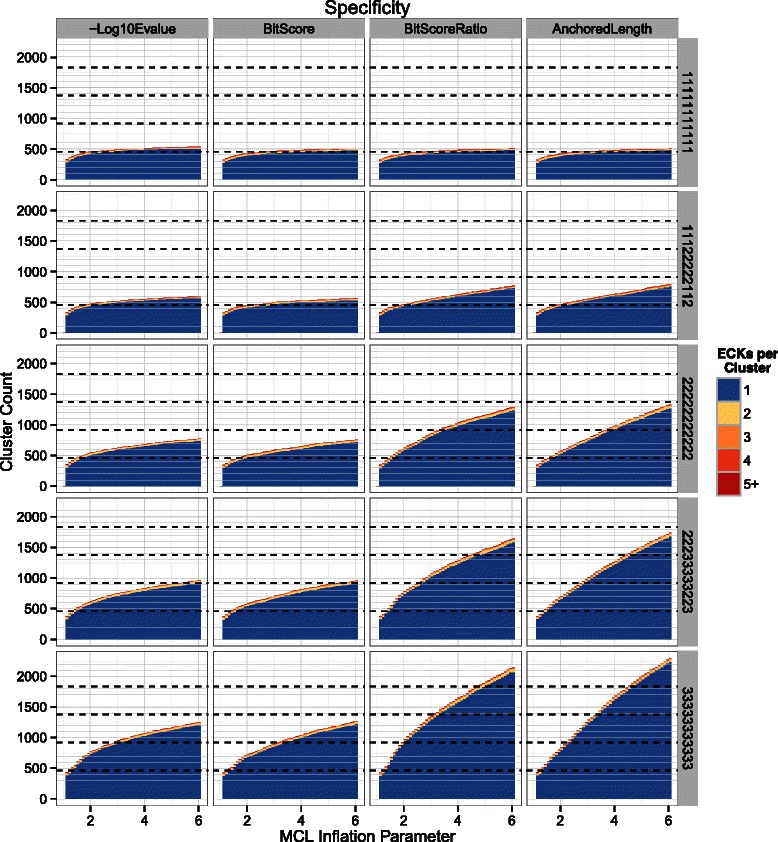
Specificity performance for each edge-weighting metric and fragmentation scenario. Specificity performance of MCL on graphs weighted using each of the four metrics (columns) over a range of inflation parameter values (x-axes) in a variety of fragmentation scenarios (rows). Vertically stacked bars indicate the number of unique ECKs from which the members of a particular MCL cluster originated. Blue segments represent MCL clusters that contain sequences from only a single ECK. Other segments represent MCL clusters containing sequences from two or more ECKs, with redder color indicating higher degrees of contamination. A large number of “pure” clusters containing only a small portion of a particular ECK can appear desceptively good, so multiples of the desired 458 total clusters are indicated with dashed horizontal lines. Simulated fragmentation scenarios are as described in Fig. 5

An initial performance comparison was made across all metrics using full-length sequences. Performance was measured in two ways that respectively evaluate the sensitivity (Fig. 5) and specificity (Fig. 6) for each metric across a range of MCL inflation parameter values. The MCL inflation parameter affects the granularity of the clusters, with larger values leading to smaller clusters. Popular values for inferring sequence homology are around 1.5, although any value larger than 1.0 should lead to convergence. Sensitivity was measured as the number of clusters into which the members of a particular ECK have been split, and specificity as the number of unique ECKs to which the members of a particular MCL cluster belong. There are a total of 458 ECKs, so a perfect score by either metric is 458 MCL clusters, each containing all sequences for a single ECK. When all sequences are intact, the performance of the various metrics is nearly identical (Figs. 5 & 6, top row). Close inspection reveals that the NLE is slightly less sensitive than the other metrics for this data set, although the difference does not seem significant. In contrast, dramatic differences emerged once some of the sequences were split into subsequences (Figs. 5 & 6, lower rows).

**Fig. 7:**
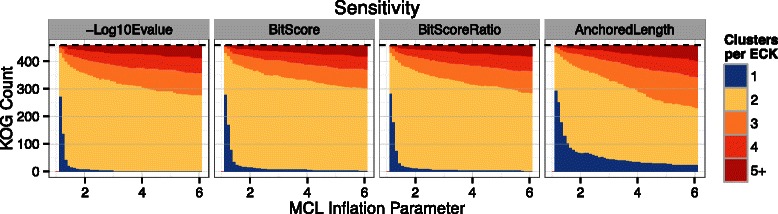
Clustering sensitivity comparison when all ECK sequences were split into even halves (scenario 22222222222 “even”). Plots are otherwise as described in Fig. 5

The beneficial effects of the BAL normalization on clustering sensitivity can be seen when all of the sequences are split into fragments of equal size (Fig. 7; Additional file 1: Figure S4). For instance, when the sequences are all split into halves, many of the break points line up, preventing any alignable overlaps and ensuring undesireable subclustering. Due to the sequence length variation within some ECKs (Additional file 1: Figure S5), however, a few BLASTP hits do connect otherwise disperate subclusters, and the BAL metric was able to successfully preserve many of these critical edges. Unfortunately, when the breakpoints are randomized and longer overlaps become more common, the benefits from these few rescued edges are quickly overshadowed by an unintended negative side-effect of the normalization (Figs. 5 & 6, lower rows).

**Fig. 8:**
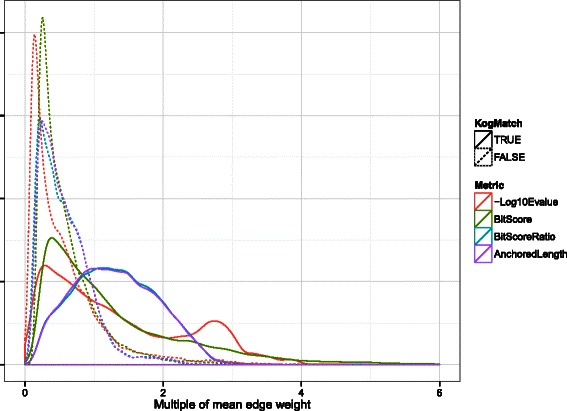
Distributions of intra- and inter-ECK edge weights by metric. Probability density plots for all four metrics, scaled by their respective mean edge weights. Each distribution has been split into intra- and inter-ECK distributions

Bit scores generated by high-quality alignments between full-length sequences are commonly 1–2 orders of magnitude greater than high-quality alignments between small sequence fragments or short overlapping regions shared by long sequences. A comparable differential in bit scores can also be seen between high- and low-quality full-length alignments, and this dynamic range turns out to be critical to the success of MCL-based homology inference. While the BSR and BAL do help to differentiate between the distributions of high-quality short alignments and low-quality long alignments, they also tighten the overall distribution by down-weighting the heaviest edges (Fig. 8). In doing so, these metrics make it less obvious to MCL that these exceptionally good alignments should be kept.

The most important discovery from this study is the observation that the bit score performs as well or better than all other metrics in all conditions we tested. Considering the practical benefits of using the bit score [11], it appears to be the best choice between the two metrics currently in popular use, and not improved by either of the other two normalization methods (BSR, BAL) considered here.

### Inter-organism normalization

One of the principle improvements introduced by OrthoMCL over TribeMCL was inter-species normalization. After a graph has been created and all missing E-values have been replaced using their heuristic, OrthoMCL computes the average edge weight over the entire graph, and also for the set of edges between each pair of organisms. It then multiplies each edge by a ratio of the average edge weight between the two corresponding organisms over the average edge weight for the entire graph. This has the effect of increasing edge weights between organisms whose sequences are more divergent in sequence space, while decreasing edge weights between organisms that are relatively close.

For each test data set, we generated a complete set of graphs both before and after inter-organism normalization. The effect was positive but minimal in all test cases evaluated for this study. Supplemental Figure S6 & S7 (Additional File 1) show the effects for full-length sequence clustering. The decision to include the additional organisms annotated by the CEGMA developers was based primarily on a desire to better demonstrate the effect of inter-organism normalization. It is possible that such normalization could significantly improve performance in certain cases, although our results demonstrate that it’s not always worth the trouble.

In cases where normalization is desired, it is not necessary to use complex or highly optimized software. We demonstrate this with our own Python program Porthos, which uses only standard modules and accomplishes the task with fewer than 100 lines of code, including the help menu. Despite this simplicity, Porthos is able to cluster all 60,758 protein sequences from the complete KOG database (2,572,291 best BLASTP hits) in less than one minute and requiring less than half a gigabyte of RAM. We present Porthos as both a simple, portable alternative to some of the more complex programs and as a heavily-commented example for anyone seeking to incorporate similar functionality into their own projects.

## Conclusions

In every scenario evaluated in this study, the bit score performed as well or better than the other three edge-weighting metrics for MCL-based inference of protein sequence homology. The significance of this finding lies in the practical benefits of using the bit score over the alternative metrics, which all require extension and/or postprocessing of tab-delimited BLAST results. We further observed little benefit from inter-organism normalization, indicating that an MCL-readable graph file created by simply extracting the sequence identifier and bit score columns from a standard tab-delimited BLAST output file could produce results comparable to those obtained from the popular OrthoMCL program.

## Methods

### Test database creation

The 458 Eukaryotic Orthologous Groups (KOGs) used to create v2.5 of the Conserved Eukaryotic Genes Mapping Approach (CEGMA) clusters (CEGs) were extracted in their entirety from the complete KOG database in order to recover the inparalogs removed by the CEGMA developers. Protein sequences from four additional organisms (*Anopheles gambiae*, *Chlamydomonas reinhardtii*, *Ciona intestinalis* and *Toxoplasma gondii*) that were annotated for an unpublished version of the CEGMA database (http://korflab.ucdavis.edu/datasets/cegma/) were then added to these 458 KOGs to increase the taxonomic diversity. None of these four organisms contributed more than a single sequence to any cluster, and none contributed sequences to all 458 clusters. We refer to the resulting 458 clusters as Expanded CEGMA KOGs (ECKs). Nearly all analyses in this study were carried out with these ECKs, although a few were repeated using the CEGMA and/or KOG databases.

### Analysis pipeline

A central Bash script called eckPipeline.sh was developed to streamline our analysis pipeline. The pipeline has 5 major steps: 1) sequence fragmentation, 2) sequence alignment, 3) graph creation, 4) sequence clustering, 5) generation of supplemental files and summary statistics.

### Sequence fragmentation

A pre-formatted sequence database and user-defined fragmentation scheme are passed to the eckTestData.py Python program, which applies the scheme to the input database to generate a set of test databases that simulate fragmentation of the sequences. Each fragmentation scheme is encoded as an integer, with each digit being mapped to an organism in alphabetical order. If the integer does not contain at least one digit for each organism represented in the sequence database, it is repeated until it reaches or exceeds this threshold, then truncated as needed. The fragmentation schemes used in this study were encoded as eleven-digit integers because the ECK database contains sequences from eleven organisms (Table 1).

Each organism contributed a different number of sequences to the ECK database, so in order to simulate half of the sequences in the database being fragmented into a certain number of pieces, it was not sufficient to simply use the first or last half of the organisms. No combination of organisms perfectly divides the database into equal halves containing exactly 3,420 sequences in each, although a combination of the sequences from *A. gambiae*, *A. thaliana*, *C. elegans*, *S. cerevisiae*, and *S. pombe* gets very close with a total 3,426. Thus, the fragmentation scheme that simulates half of the sequences being split into two pieces was encoded as 11122222112, and the scheme that simulates half being split into two pieces and the other half split into three was encoded as 22233333223.

From this mapping, the pipeline generates two pairs (a total of four) test data sets. In the “ordered” pair, these digits directly determine the number of fragments into which the sequences from a particular organism will be split. In the “shuffled” pair, the integer labels are first mapped to the sequences within each ECK cluster, but then they are shuffled before fragmentation (Fig. 2). Within each pair of data sets, the “even” set uses evenly distributed breakpoints within each sequence, while in the “random” set they are randomly distributed.

The format of the sequence database is described in the GitHub wiki.

### Sequence alignment

Each of the four data sets generated in the first step are formatted as BLASTP databases and then aligned against themselves using BLASTP v.2.2.28+ with an E-value cutoff of 1e-5 (−evalue 1e-5) and soft masking turned on (−soft_masking true). The output is formatted as a tab-delimited table with headers and two extra columns for the query and subject sequence lengths (−outfmt ‘7 std qlen slen’).

### Graph creation

Graph creation is accomplished with the blast2graphs.py Python program, which converts a table of BLAST hits, with or without header lines, into a set of eight graphs corresponding to the four edge-weighting metrics used in this study, both before and after inter-organism normalization. All metrics use only the best hit for each pair of aligned sequences.

Normalization is accomplished by calculating, for each metric, the average edge weight for the entire graph, and the average weight for all edges connecting sequences from each pair of organisms. Each edge is then multiplied by a ratio of the average graph edge weight over the average weight between the corresponding organisms.

### Sequence clustering

For each graph, MCL (v12-068) is used to generate clusters with inflation parameter values ranging from 1.1 to 6.0, creating a total of 50 clusterings per graph.

### Supplemental files and summary statistics

The mcl2rtab.py Python program is used to generate a pair of files containing summary statistics for all 400 clusterings corresponding to the eight graphs generated from a single BLASTP file. These files are then converted into stacked barcharts using ggplot2 in R with the barcharts.R program. One last custom Python program called graphs2gml.py is used to generate annotated representations of the graphs and clusterings in a variety of popular file formats that can be read into interactive graph visualization software, such as Cytoscape [30, 31].

### Availability of supporting data

All custom software used in this study can be cloned from a dedicated github repository: https://github.com/trgibbons/BlastGraphMetrics.git. Instructions for installation and execution are included in the project wiki.

The ECK test database was derived from the publicly available CEGMA and KOG databases. The CEGMA database can be downloaded from the Korf lab website: http://korflab.ucdavis.edu/datasets/cegma/. The KOG database can be downloaded from the National Center for Biotechnology Information website: ftp://ftp.ncbi.nih.gov/pub/COG/KOG. The downloadEckDatabase.py program can also be used to automatically fetch and format all three databases from their respective websites.

Our simple Python orthology inference program Porthos can be cloned from a separate dedicated github repository: https://github.com/trgibbons/porthos.git.

## Additional file

Additional file 1:
**Sensitivity performance comparison for each test database.**

## Abbreviations

MCL: Markov CLustering
COG: Clusters of Orthologous Groups
KOG: euKaryotic Orthologous Groups
CEGMA: Conserved Eukaryotic Genes Mapping Approach
ECK: Expanded CEGMA KOGs
SBS: Self Bit Score
BSR: Bit Score Ratio
AL: Anchored alignment Length
BAL: Bit score over Anchored alignment Length
E-value: Expectation value
NLE: Negative common (base ten) Log of the Expectation value
