# Erratum to: Evaluation of BLAST-based edge-weighting metrics used for homology inference with the Markov Clustering algorithm

**DOI:** 10.1186/s12859-015-0690-1

**Published:** 2015-08-28

**Authors:** Theodore R. Gibbons, Stephen M. Mount, Endymion D. Cooper, Charles F. Delwiche

**Affiliations:** 1 Department of Cell Biology and Molecular Genetics, University of Maryland, College Park, Maryland 20742 USA; 2 Center for Bioinformatics and Computational Biology, University of Maryland, College Park, Maryland 20742 USA; 3 Maryland Agricultural Experiment Station, University of Maryland, College Park, Maryland 20742 USA

## Erratum

Unfortunately, [1] was published without incorporation of all requested corrections. The correct equation for computing the E-value from a bit score is

$$ \mathrm{E}\hbox{-} \mathrm{value}=\frac{mN}{2^{BS}}, $$

not

$$ E\mathit{\hbox{-}} value=\frac{mN}{2_{BS}}. $$

The image file used for Figure 2 was incorrect. The correct figure is provided here as Fig. 1. The name of Additional File 1 should be “Supplemental Figures”, not “Sensitivity performance comparison for each test database.” The University of Maryland, College Park is located in College Park, Maryland, not College Park, Baltimore, Maryland. The correct addresses are provided in this erratum.

**Fig. 1:**
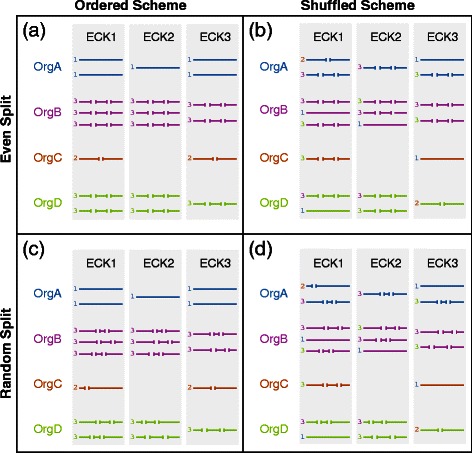
Illustration of simulated sequence fragmentation. This example illustrates the four different ways in which the fragmentation scheme 1323 would be applied to a toy input test database with only three clusters containing sequences from only four organisms. The four resulting test sets represent a cross of two variables, arranged here into rows and columns. The sequences in the top row (**a & b**) have been split into even subsequences. The sequences in the lower row (**c & d**) have been randomly fragmented into uneven subsequences. In the left column (**a & c**), the user-defined integer assigned to each organism directly determines the number of subsequences into which each sequence is split. In the right column (**b & d**), these integers are first mapped to all sequences within a cluster, but are then shuffled within that cluster before fragmentation
