# Supplementary material for: Evaluation of BLAST-based edge-weighting metrics used for homology inference with the Markov Clustering algorithm
Source: BMC Bioinformatics. 2015 Jul 10;16:218. doi: 10.1186/s12859-015-0625-x (PMC4496851; doi:10.1186/s12859-015-0625-x)
Supplement: Additional file 1: — Sensitivity performance comparison for each test database. [file 12859_2015_625_MOESM1_ESM.pdf]

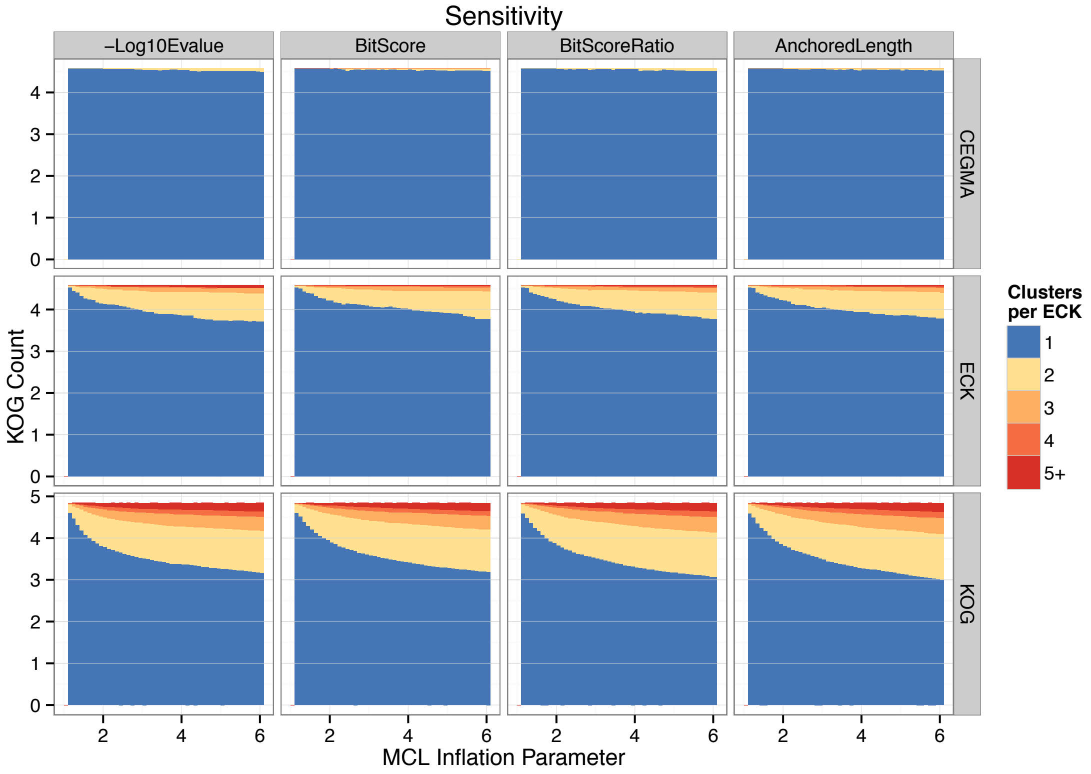

Supplemental Figure 1 – Sensitivity performance comparison for each test database
Sensitivity performance on CEGMA (upper row), ECK (middle row), and KOG (lower row) databases. The sensitivity problems observed with the KOG database are not observed with the CEGMA database, but are restored with the ECK database. Plots are otherwise as described in Figure 5.

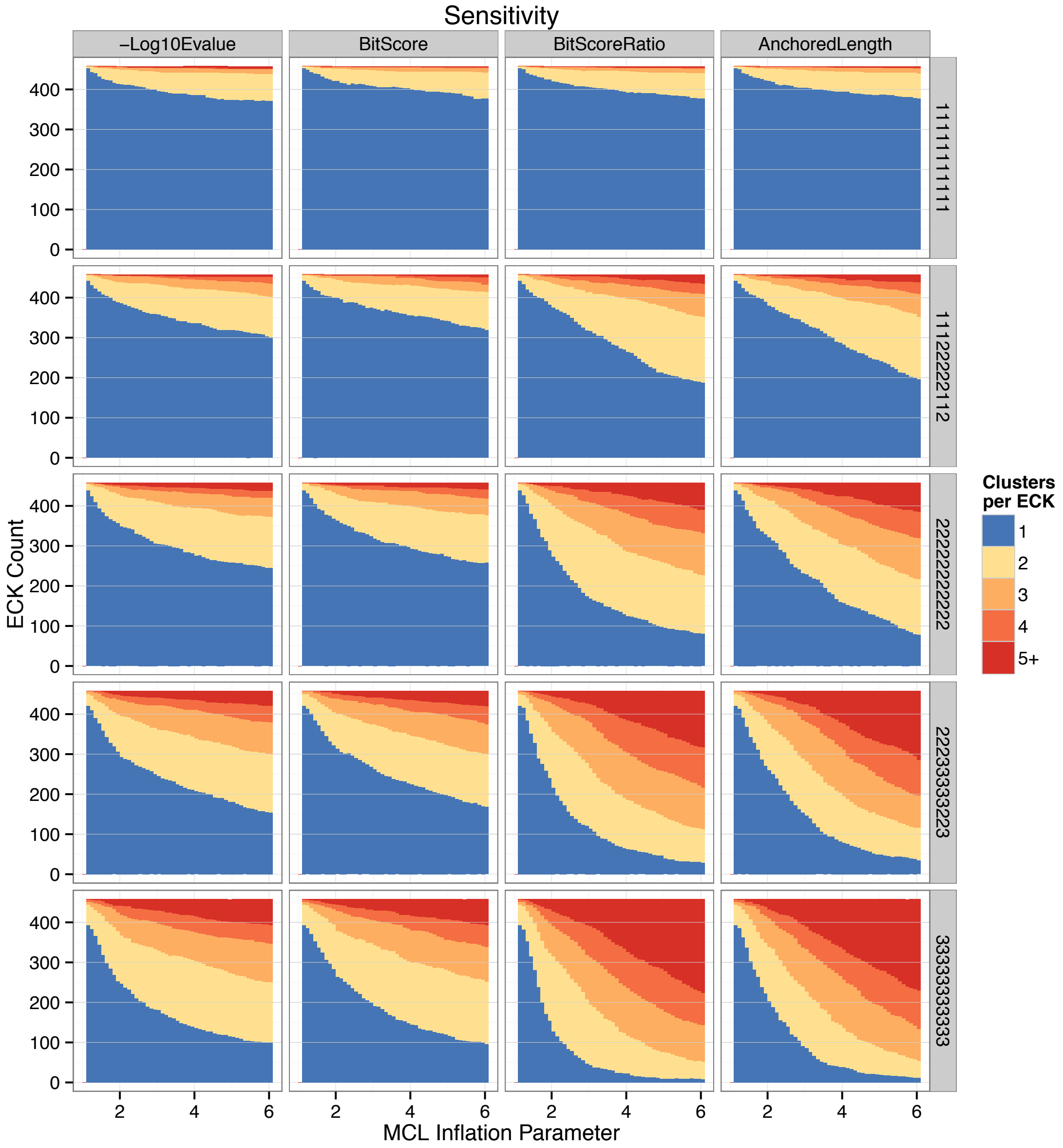

Supplemental Figure 2 – Sensitivity performance comparison with ordered application of the fragmentation scheme
Plots were prepared identically to those in Figure 5, except that the fragmentation scheme was applied directly along organismal lines. Results are indistinguishable from those shown in Figure 5, indicating that no organism's sequences were more or less important to clustering.

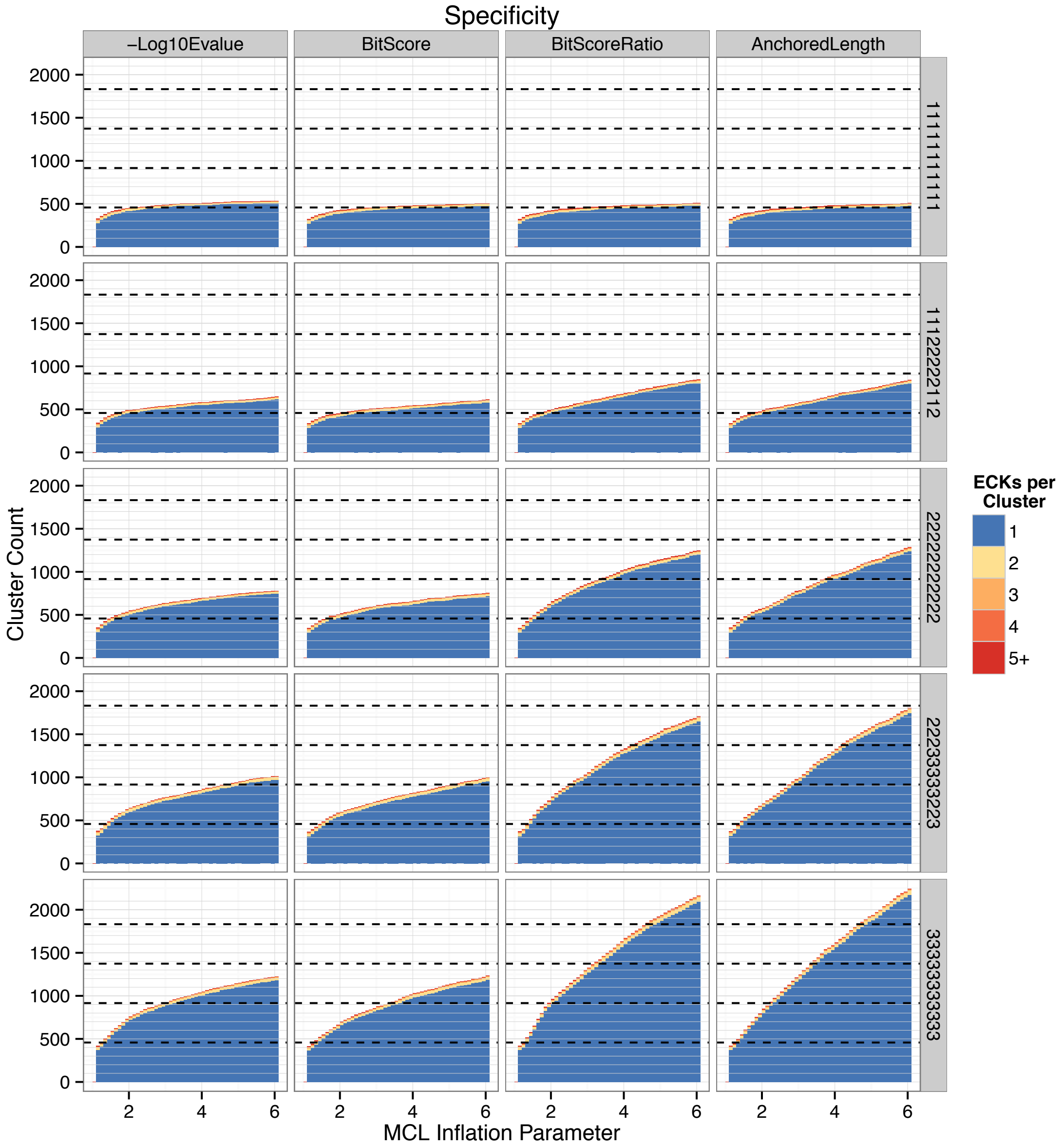

Supplemental Figure 3 – Specificity performance comparison with ordered application of the fragmentation scheme
Plots were prepared identically to those in Figure 6, except that the fragmentation scheme was applied directly along organismal lines. Results are indistinguishable from those shown in Figure 6, indicating that no organism's sequences were more or less important to clustering.

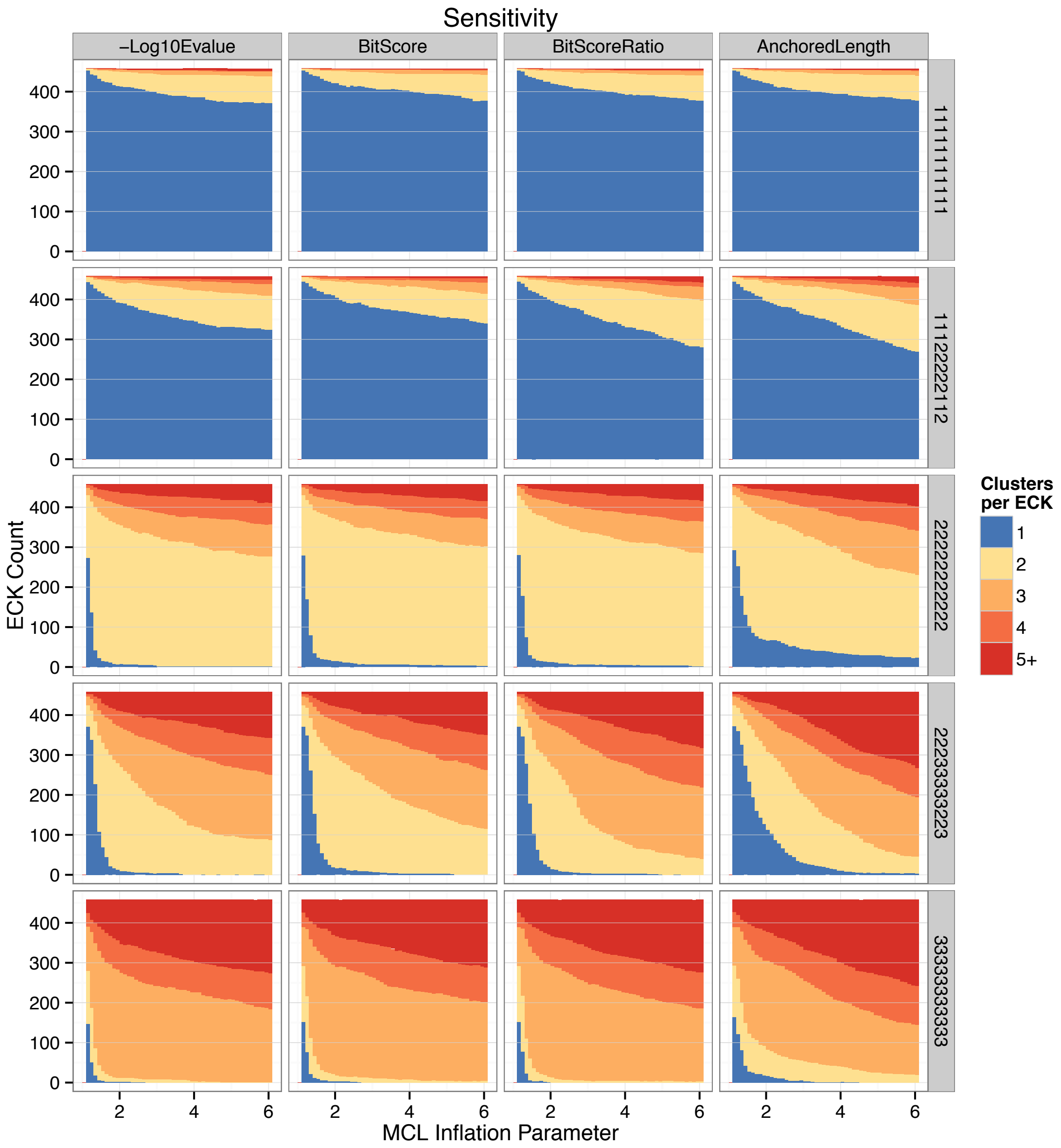

Supplemental Figure 4 – Sensitivity performance comparison with evenly fragmented sequences
Plots were prepared identically to those in Figure 5, except that sequences were fragmented in equal pieces (ie. halves or thirds). Results confirm that when all sequences are split into equal pieces, many breakpoints align, leaving minimal overlapping sequences. The consequent lack of high quality edges between halves or thirds of sequences makes it nearly impossible to recover complete clusters containing all fragments. The bit score over anchored alignment length (BAL) metric performs slightly better than they other metrics in this scenario as a result of successfully strengthening whatever weak edges do manage to cross these fragmentation boundaries.

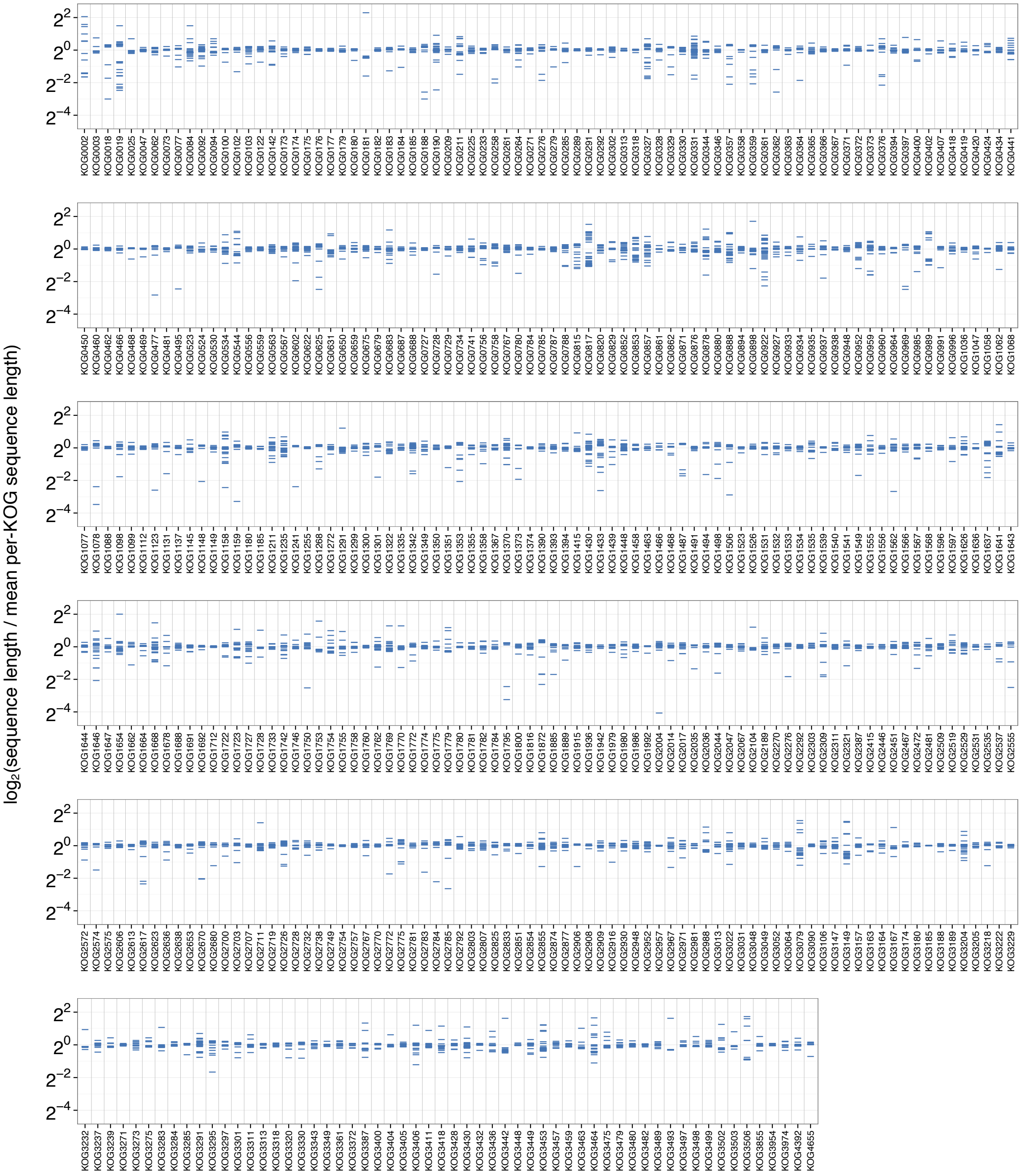

Supplemental Figure 5 – Sequence lengths relative to the mean for each ECK

Sequence lengths divided by the mean length for each ECK (blue dashes). Sequences within the ECK database are organized using KOG identifiers, and are therefore sorted by these KOG identifiers along the x-axis. The y-axis has been transformed into a $\log_2$ scale to emphasize fold-changes relative to the mean sequence lengths. Most ECKs have very uniform distributions of sequence lengths, but some contain sequences with lengths varying from 1/8 to 4x the corresponding mean. These differences provide an important variety of test cases when splitting the sequences evenly because they prevent BLAST-alignable overlaps within most ECKs, but not all.

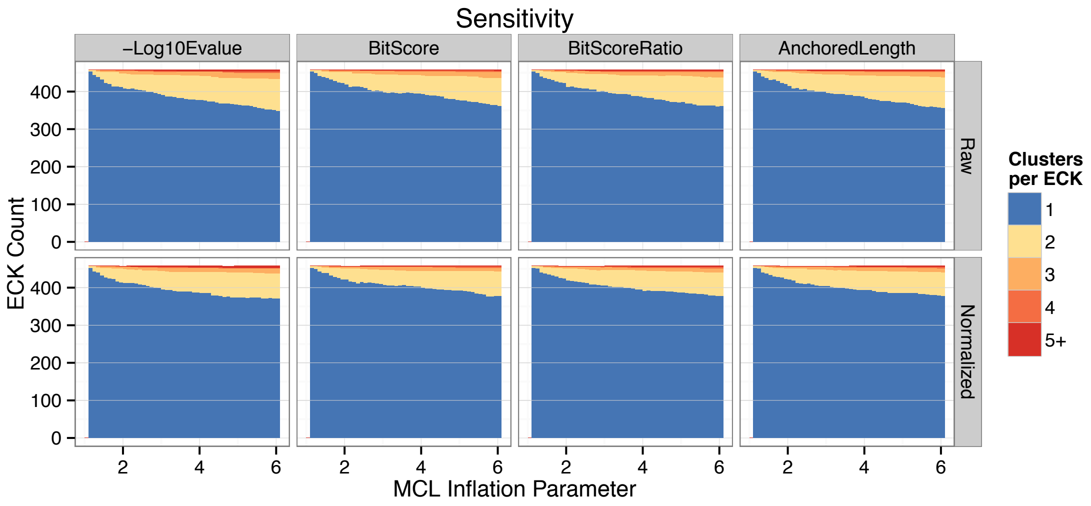

Supplemental Figure 6 – Sensitivity performance with and without inter-organism normalization
Sensitivity performance on the ECK database with all sequences intact before (upper row) and after (lower row) inter-organism normalization. Plots are otherwise as described in Figure 5. Close inspection reveals some small improvement from the normalization.

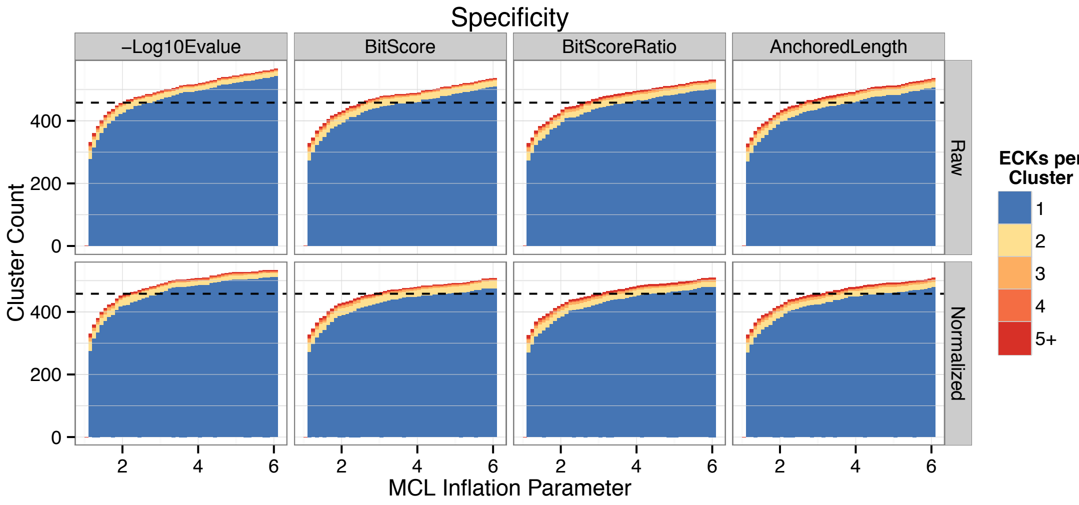

Supplemental Figure 7 – Specificity performance with and without inter-organism normalization
Specificity performance on the ECK database with all sequences intact before (upper row) and after (lower row) inter-organism normalization. Plots are otherwise as described in Figure 6. Close inspection reveals some small improvement from the normalization.
